# Invasive Infections with Multidrug-Resistant Yeast *Candida auris*, Colombia

**DOI:** 10.3201/eid2301.161497

**Published:** 2017-01

**Authors:** Soraya E. Morales-López, Claudia M. Parra-Giraldo, Andrés Ceballos-Garzón, Heidys P. Martínez, Gerson J. Rodríguez, Carlos A. Álvarez-Moreno, José Y. Rodríguez

**Affiliations:** Universidad Popular del Cesar, Valledupar, Colombia (S.E. Morales-López, H.P. Martínez);; Pontificia Universidad Javeriana, Bogotá, Colombia (C.M. Parra-Giraldo, A. Ceballos-Garzón);; Centro de Investigaciones Microbiológicas del Cesar (CIMCE), Valledupar, Colombia (G.J. Rodríguez, J.Y. Rodríguez);; Clínica Laura Daniela, Valledupar (G.J. Rodríguez, J.Y. Rodríguez);; Universidad Nacional de Colombia, Bogotá (C.A. Álvarez-Moreno);; Clínica Universitaria Colombia, Colsanitas, Colombia (C.A. Álvarez-Moreno)

**Keywords:** *Candida auris*, Americas, antimicrobial resistance, candidemia, VITEK, Etest, Colombia, fungemia, fungi

## Abstract

*Candida auris* is an emerging multidrug-resistant fungus that causes a wide range of symptoms. We report finding 17 cases of *C. auris* infection that were originally misclassified but correctly identified 27.5 days later on average. Patients with a delayed diagnosis of *C. auris* had a 30-day mortality rate of 35.2%.

*Candida auris* is an emerging multidrug-resistant fungus that causes a wide range of infections that are sometimes associated with high mortality rates (*1*–*4*)*. C. auris* was first isolated in Japan and described as a new species in 2009 (*5*). In 2011, it was described as a cause of fungemia in South Korea (*4*) and was later isolated from patients in India (*2*), South Africa (*6*), Kuwait (*3*), and Venezuela (*1*).

We report 17 clinical isolates of *C. auris* recovered from 17 patients hospitalized in 6 institutions in the northern region of Colombia from February through July 2016. We reviewed patient medical records; analyzed microbiological, demographic, and clinical variables; and evaluated the mortality rate 30 days after yeast isolation. The initial pathogen identification was made with the method available at each institution: VITEK 2 system (bioMérieux, Marcy l’Étoile, France); Phoenix (Becton Dickinson, Franklin Lakes, New Jersey, USA); MicroScan AutoSCAN 4 and MicroScan Walkaway (Beckman Coulter, Brea, California, USA); and API Candida (bioMérieux) (Table). Given the unusual prevalence of *C. haemulonii* and discordance in the micromorphologic characteristics of some isolates, we cultured strains in CHROMagar Candida medium (CHROMagar Candida, Paris, France) and identified them by using matrix-assisted laser desorption/ionization time-of-fight (MALDI-TOF) mass spectrometry (Bruker Daltonik, Bremen, Germany). All the isolates showed pink colonies on CHROMagar Candida medium and were identified as *C. auris* by MALDI-TOF mass spectrometry (scores >2.0). 

**Table:** Identification and antifungal susceptibilities of *Candida auris* clinical isolates of six hospitals, northern region of Colombia, 2016*

Isolate ID	Hospital no.	Specimen origin	Biochemical identification (system)	Pre-AFT	VITEK cards						Etest/AMB	
FLC	MCF	CAS	VRC	AMB		24 h	48 h
001	1	Blood	*C. haemulonii* (VITEK)	None	16	0.12	<0.25	0.25	8		0.75	1
002	1	CSF	*C. tropicalis* (MicroScan Walkaway)	CAS	16	0.12	<0.25	<0.12	8		0.75	1
003	1	Blood	*C. famata* (API Candida)	FLC	16	<0.06	<0.25	<0.12	8		1	1
004	5	Blood	*C. haemulonii* (Phoenix)	FLC	16	0.12	<0.25	0.25	>16		1	1.5
005	2	Blood	*C. haemulonii* (VITEK)	FLC	>64	0.25	0.5	2	>16		1	1.5
006	4	Blood	*C. haemulonii* (VITEK)	None	>64	0.12	<0.25	0.5	8		1	1
007	3	Peritoneal fluid	*C. albicans* (MicroScan autoSCAN)	FLC	16	0.12	<0.25	0.25	8		1	1.5
008	2	Blood	*C. haemulonii* (VITEK)	FLC	16	0.12	<0.25	0.25	8		0.38	0.75
009	1	Blood	*C. tropicalis* (MicroScan Walkaway*)/ C. famata* (API Candida)	FLC, CAS	32	0.12	<0.25	0.25	8		2	3
010	5	Bone	*C. haemulonii* (VITEK)	FLC, AFG, CAS	16	0.12	<0.25	0.25	8		0.75	1
011	6	Urine	*C. haemulonii* (Phoenix)	None	32	0.12	<0.25	0.25	8		1.5	1.5
012	3	Blood	*C. albicans* (MicroScan AutoSCAN)	FLC	32	0.12	<0.25	1	8		1	2
013	3	Blood	*C. haemulonii* (VITEK)	None	32	0.12	<0.25	1	8		0.75	2
014	3	Blood	*C. haemulonii* (VITEK)	FLC	>64	0.12	<0.25	2	8		1.5	1.5
015	2	Blood	*C. haemulonii* (VITEK)	None	32	0.12	<0.25	0.25	8		0.75	2
016	2	Blood	*C. haemulonii* (VITEK)	FLC	>64	0.12	<0.25	2	>16		1	2
017	4	Blood	*C. haemulonii* (VITEK)	CAS	>64	0.12	<0.25	2	>16		2	4
MIC (mg/L) range					16 to >64	<0.06 to 0.25	<0.25 to 0.5	<0.12 to 2	8 to >16		0.38 to 2	0.75 to 4
MIC_50_					32	0.12	<0.25	0.25	8		1	1.5
MIC_90_					>64	0.12	<0.25	2	>16		2	2

*AFG, anidulafungin; AMB, amphotericin B; CAS, caspofungin; CSF, cerebrospinal fluid; FLC, fluconazole; ID, identification; MCF, micafungin; MIC_50_, minimal inhibitory concentration for 50% of yeast; MIC_90_, minimal inhibitory concentration for 90% of yeast; Pre-AFT, use of antifungal therapy before the isolation of *C. auris*; VRC, voriconazole.

We tested yeast isolates for in vitro susceptibility by VITEK cards (caspofungin, micafungin, fluconazole, voriconazole, and amphotericin B). Additionally, we performed the agar diffusion method using Etest strips (bioMérieux, France) to amphotericin B according to the manufacturer’s instructions. *C. parapsilosis* ATCC 22019 was used as the control. The range, mode, MIC_50_, and MIC_90_ were calculated.

Of the 17 patients, 9 were men (52.9%); age range was 0–77 years (median 36 years). Fifteen (88.2%) were hospitalized in intensive care units and 2 in medical wards; no patients were transferred between hospitals. 

Blood samples from 13 (76.4%) patients showed fungemia; for the remaining 4, *C. auris* was isolated from peritoneal fluid, cerebrospinal fluid, bone, or urine (Table). Most patients had a central venous catheter (n = 16, 94.1%), a urinary catheter (n = 15, 88.2%), and mechanical ventilation (n = 10, 58.8%). Additionally, some had risk factors described previously for candidemia: erythrocyte transfusion (n = 12, 70.5%), parenteral nutrition (n = 8, 47%), abdominal surgery (n = 7, 41.1%), hemodialysis (n = 5, 29.4%), diabetes (n = 3, 17.6%), pancreatitis (n = 2, 11.7%), cancer (n = 2, 11.7%), and HIV infection (n = 1, 5.8%).

The average number of days from hospitalization to isolation of *C. auris* was 27.5 days (SD ± 19.9 days). Before the isolation of *C. auris*, 15 (88.2%) and 12 (70.6%) patients received broad-spectrum antimicrobial therapy and antifungal therapy, respectively. Of the latter, 8 received fluconazole, 2 caspofungin, 1 fluconazole and caspofungin, and 1 fluconazole, caspofungin, and anidulafungin (given at a different time periods) (Table). The time from isolation of *C. auris* to the initial application of effective antifungal therapy averaged 3.7 days. The 30-day mortality rate in all patients and in those who had fungemia was 35.2% and 38.4%, respectively.

*C. auris* is phylogenetically related to *C. haemulonii*, for which it is often mistaken with the methods currently used for identification (*2*–*6*). Our isolates were originally misidentified as *C. haemulonii*, *C. famata*, *C. albicans*, or *C. tropicalis*, depending on the method used in the hospital. The identification of isolates by MALDI-TOF mass spectrometry has also been described in the literature as an adequate and fast method for identifying *C. auris* (*7*).

Because the Clinical and Laboratory Standards Institute does not currently provide breakpoints for *C. auris*, no categorical interpretation of results is available; thus, only the MICs obtained for antifungal drugs tested in our study were indicated (Table). Although misleading, elevated MICs of amphotericin B by VITEK card have been previously described (*7*); this study also found discrepancies with Etest strips, which could lead to the selection of inappropriate therapy if only 1 method is used.

The presence of *C. auris* in these patients has clinical and epidemiologic implications, considering the associated mortality rate confirmed in this report and the absence of sufficient technology in clinical laboratories both to confirm their identification and to carry out testing for antifungal susceptibility. The lack of suitable diagnostics complicates patient treatment and changes on the empiric treatment of invasive *Candida* spp. infections are needed.

Our data contributes to the knowledge of the epidemiology of this species at a regional level. Although we had already reported *Candida* spp. in Colombia (*8*), no information regarding these species on the Caribbean coast is available. Given the association of *Candida* spp. with outbreaks in hospitals, according to the Centers for Disease Control and Prevention, it is necessary to further strengthen measures for fungal infection control to prevent possible spread.
